# Supplementary material for: Visual Integration of Genome-Wide Association Studies and Differential Expression Results with the Hidecan R Package
Source: Genes (Basel). 2024 Sep 25;15(10):1244. doi: 10.3390/genes15101244 (PMC11506992; doi:10.3390/genes15101244)
Supplement: Supplementary file 1 [file genes-15-01244-s001.zip › genes-3208002-Supplementary_material_2_examples.pdf]

Visual integration of GWAS and differential expression results with the hidecan R package

Supplementary Material 2 – Examples

O. Angelin-Bonnet, M. Vignes, P. J. Biggs, S. Baldwin, S. Thomson

```
library(hidecan)
library(GWASpoly)
library(ggplot2)
```

Example 1 – potato tuber bruising

The data

The dataset used in this example is presented in (Angelin-Bonnet et al. 2023). In this study, tetraploid potato plants from a half-sibling breeding population were used to assess the genetic components of tuber bruising. Capture sequencing was used to obtain genomic information about the individuals, and a genome-wide association study (GWAS) was performed on 72,847 genomic biallelic variants obtained from 158 plants for which a bruising score was measured. The GWAS analysis was carried with the `GWASpoly` package.

In addition, expression data were obtained for 25,163 transcribed genes, and a differential expression (DE) analysis was carried out between 41 low- and 33 high-bruising samples.

Finally, a literature search yielded a list of 42 candidate genes identified in previous studies as involved in potato tuber bruising mechanisms.

A subset of the GWAS and DE results, as well as the list of candidate genes from the literature, are made available in the `hidecan` package through the `get_example_data()` function. From the complete GWAS results table, half of the genomic variants with a GWAS score < 3.5 were randomly selected and consequently discarded, yielding a dataset with GWAS scores for 35,481 variants. Similarly, half of the transcribed genes in the DE results table with an adjusted p-value > 0.05 were randomly selected and discarded, yielding a dataset with DE results for 10,671 transcribed genes. This filtering was performed to reduce the size of the datasets (in accordance with CRAN policies), but ensures that all significant markers and genes are retained in the datasets. Finally, some of the candidate genes located on chromosome 3 were

removed from the example dataset for better clarity in the resulting HIDECAN plot, leaving 32 candidate genes.

The example data can be obtained with:

```
data <- get_example_data()

str(data, max.level = 1)
```

```
List of 3
 $ GWAS: tibble [35,481 x 4] (S3: tbl_df/tbl/data.frame)
 $ DE  : tibble [10,671 x 7] (S3: tbl_df/tbl/data.frame)
 $ CAN : tibble [32 x 6] (S3: tbl_df/tbl/data.frame)
```

The `get_example_data()` function returns a list of 3 data-frames: the GWAS results table (`GWAS` element), the DE results table (`DE` element) and the candidate genes table (`CAN` element).

The GWAS results table contains, for each genomic variant:

- its genomic position (chromosome and physical position in bp) on the potato genome (PGSC-DM v4.03);
- its GWAS score (i.e. $-\log_{10}$(p-value)).

```
head(data[["GWAS"]])
```

```
# A tibble: 6 x 4
  id                   chromosome position score
  <chr>                <chr>         <dbl> <dbl>
1 ST4.03ch00_45467783 ST4.03ch00 45467783 0.191
2 ST4.03ch01_88589716 ST4.03ch01 88589716 1.84
3 ST4.03ch02_48614228 ST4.03ch02 48614228 0.381
4 ST4.03ch03_62263578 ST4.03ch03 62263578 0.661
5 ST4.03ch04_72139135 ST4.03ch04 72139135 0.640
6 ST4.03ch05_52040302 ST4.03ch05 52040302 0.346
```

The DE results table contains, for each transcribed gene:

- its genomic position (i.e. chromosome, as well as start and end positions in bp);
- its adjusted p-value and log2-fold change from the differential expression analysis;
- a label describing the function of the gene (ignored by `hidecan`).

```r
head(data[["DE"]])
```

```
# A tibble: 6 x 7
  gene               chromosome   padj log2FoldChange    start      end label
  <chr>              <chr>       <dbl>          <dbl>    <dbl>    <dbl> <chr>
1 PGSC0003DMG400032056 ST4.03ch00  0.787       0.0114   45813195 45813526 Prote~
2 PGSC0003DMG400018039 ST4.03ch01  0.630       0.00529  88623473 88627702 PhD-f~
3 PGSC0003DMG400020231 ST4.03ch02  0.864       0.00362  48563271 48578978 Acety~
4 PGSC0003DMG400009197 ST4.03ch03  0.530       0.0320   62256322 62258929 Phosp~
5 PGSC0003DMG403025662 ST4.03ch04  0.975       0.00225  72168842 72170119 Conse~
6 PGSC0003DMG400023316 ST4.03ch05 NA          -0.000726 52039916 52040326 Conse~
```

The candidate gene table contains, for each gene extracted from the literature:

- its genomic position (i.e. chromosome, as well as start and end positions in bp);
- its label, corresponding to a short version of the complete gene name;
- its complete gene name (ignored by `hidecan`).

```r
head(data[["CAN"]])
```

```
# A tibble: 6 x 6
  id                   chromosome    start      end name       gene_name
  <chr>                <chr>         <dbl>    <dbl> <chr>      <chr>
1 PGSC0003DMG400003155 ST4.03ch03 46757152 46762127 4CL        4-coumarate-CoA ~
2 PGSC0003DMG400014223 ST4.03ch03 57466692 57469946 4CL2       4-coumarate-CoA ~
3 PGSC0003DMG400011189 ST4.03ch07  1001854  1006278 HQT        HQT
4 PGSC0003DMG400005492 ST4.03ch05 36342746 36347409 PAL        phenylalanine am~
5 PGSC0003DMG400005279 ST4.03ch05 42523943 42525912 peroxidase peroxidase
6 PGSC0003DMG400007782 ST4.03ch03 38537202 38540209 PHO1A      PHO1A
```

Constructing the HIDECAN plot

From these three tables, a HIDECAN plot can be generated through the `hidecan_plot()` function. This requires to set a significance threshold on the GWAS score of the genomic variants, as well as on the DE score and log2-fold change of the transcribed genes, which will determine which variants and genes are represented in the plot. For this example, we will set the significance threshold for the GWAS analysis to 4 (corresponding to a p-value of 1×10^{-4}). We will use for the DE results a significance threshold of 1.3 (corresponding to an adjusted p-value of 0.05) and a log2 fold-change threshold of 0 (which amounts to no filtering based on

the log2-fold change). These thresholds are set through the `score_thr_gwas`, `score_thr_de` and `log2fc_thr` arguments, respectively.

```
hidecan_plot(
  gwas_list = data[["GWAS"]], ## data-frame of GWAS results
  de_list = data[["DE"]],     ## data-frame of DE results
  can_list = data[["CAN"]],   ## data-frame of candidate genes
  score_thr_gwas = 4,         ## sign. threshold for GWAS
  score_thr_de = 1.3,         ## sign. threshold for DE
  log2fc_thr = 0              ## log2-fold change threshold for DE
)
```

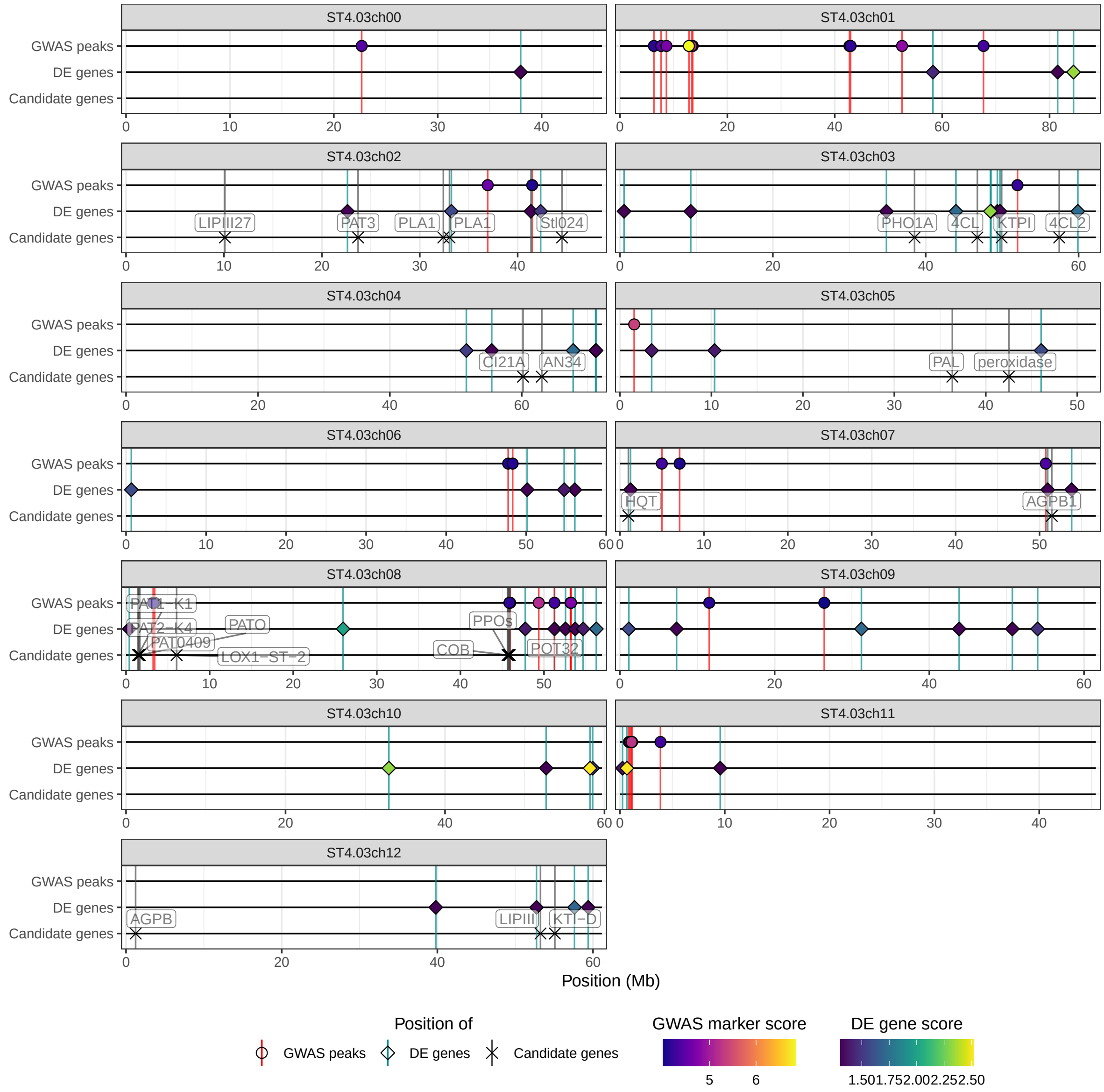

This example, as well as a demonstration of additional options available through the `hidecan_plot()` function are available online at https://plantandfoodresearch.github.io/hidecan/articles/hidecan.html.

Example 2 – GWASpoly output

The data

In this second example, we demonstrate how the output of the `GWASpoly` package can be used directly to generate a HIDECAN plot without formatting of the data. A tutorial for the `GWASpoly` package can be found at https://jendelman.github.io/GWASpoly/GWASpoly.html.

This example is based on the example dataset provided in the publication associated with the `GWASpoly` package (Rosyara et al. 2016), containing genomic and phenotypic information for 221 tetraploid potato lines from the SolCAP diversity panel. In this example, we focus on three of the recorded traits: tuber eye depth, tuber shape, and tuber sucrose content. We will test out four different genetic models for the genotype-phenotype association model: general, additive, simplex reference-dominant and simplex alternate-dominant. For details about the different genetic models, see the GWASpoly publication (Rosyara et al. 2016).

The GWAS analysis can be run with the `GWASpoly` package with the following code:

```
library(GWASpoly)

## Path to genomic and phenotype data files
genofile <- system.file("extdata", "TableS1.csv", package = "GWASpoly")
phenofile <- system.file("extdata", "TableS2.csv", package = "GWASpoly")

## Reading SolCAP data
data <- read.GWASpoly(
  ploidy = 4,
  pheno.file = phenofile,
  geno.file = genofile,
  format = "ACGT",
  n.traits = 13,
  delim = ","
)
```

```
Number of polymorphic markers: 3521
Missing marker data imputed with population mode
N = 187 individuals with phenotypic and genotypic information
Detected following fixed effects:
Grp1
Grp2
Grp3
Grp4
```

```
Detected following traits:
total_yield
chip_color
tuber_eye_depth
tuber_shape
tuber_size
tuber_length
tuber_width
sucrose
log10_glucose
log10_fructose
malic_acid
vine_maturity_95d
vine_maturity_120d
```

```r
## Computing K matrix
data.original <- set.K(
  data,
  LOCO = FALSE,
  n.core = 2
)

## Performing GWAS
gwaspoly_res <- GWASpoly(
  data.original,
  models = c("general", "additive", "1-dom"),
  traits = c("tuber_eye_depth", "tuber_shape", "sucrose"),
  n.core = 2
)
```

```
Using default value for max.geno.freq = 1 - 5/N
Analyzing trait: tuber_eye_depth
P3D approach: Estimating variance components...Completed
Testing markers for model: general
Testing markers for model: additive
Testing markers for model: 1-dom-alt
Testing markers for model: 1-dom-ref
Analyzing trait: tuber_shape
P3D approach: Estimating variance components...Completed
Testing markers for model: general
Testing markers for model: additive
Testing markers for model: 1-dom-alt
```

```
Testing markers for model: 1-dom-ref
Analyzing trait: sucrose
P3D approach: Estimating variance components...Completed
Testing markers for model: general
Testing markers for model: additive
Testing markers for model: 1-dom-alt
Testing markers for model: 1-dom-ref
```

```r
## Computing significance threshold
gwaspoly_res_thr <- set.threshold(
  gwaspoly_res,
  method = "M.eff",
  level = 0.05
)
```

```
Thresholds
                general additive 1-dom-alt 1-dom-ref
tuber_eye_depth    4.67     4.67      4.46      4.49
tuber_shape        4.67     4.67      4.46      4.49
sucrose            4.67     4.67      4.46      4.49
```

The resulting `gwaspoly_res_thr` object contains information about the genomic variants as well as their score for each of the traits and genetic models tested, and the significance threshold for each trait and genetic model used.

As an alternative to running the analysis through the code presented above, the `gwaspoly_res_thr` object is available through the `hidecan` package, and can be loaded with the following command:

```r
gwaspoly_example_file <- system.file("extdata/gwaspoly_res_thr.rda",
                                     package = "hidecan")
gwaspoly_res_thr <- readRDS(gwaspoly_example_file)
```

Constructing the HIDECAN plot

The output of the `set.threshold()` function from the `GWASpoly` package can be used as an input for the `hidecan` function `hidecan_plot_from_gwaspoly()`, in order to create a HIDECAN plot representing the GWAS results for each combination of trait and genetic model. The significance thresholds are directly read from the `GWASpoly` output object and do not need to be manually set by the user. In order to improve the clarity of the figure, the argument `remove_empty_chrom` is set to `TRUE`, which removes from the plot chromosomes with

no significant markers. The `chrom_limits` argument is used to zoom in on specific genomic regions for each chromosome (minimum and maximum positions are specified in base pairs):

```
hidecan_plot_from_gwaspoly(
  gwaspoly_res_thr,
  remove_empty_chrom = TRUE,
  chrom_limits = list(
    "5" = c(2e6, 2.5e6),
    "8" = c(53e6, 53.5e6),
    "10" = c(48.5e6, 49e6),
    "11" = c(0.5e6, 1e6)
  )
)
```

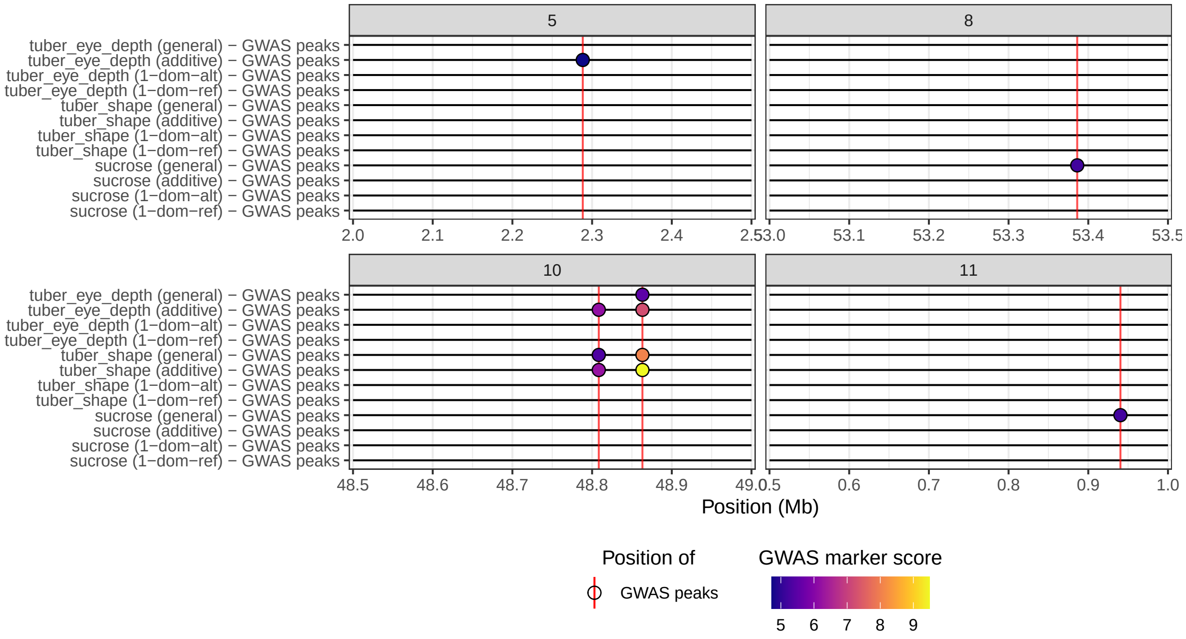

Specifying traits and/or genetic models

We can also restrict the plot to specific traits and/or genetic models through the `traits` and `models` arguments:

```
hidecan_plot_from_gwaspoly(
  gwaspoly_res_thr,
  traits = "tuber_eye_depth",
```

```
  models = c("general", "additive"),
  remove_empty_chrom = TRUE,
  chrom_limits = list(
    "5" = c(2e6, 2.5e6),
    "10" = c(48.5e6, 49e6)
  )
)
```

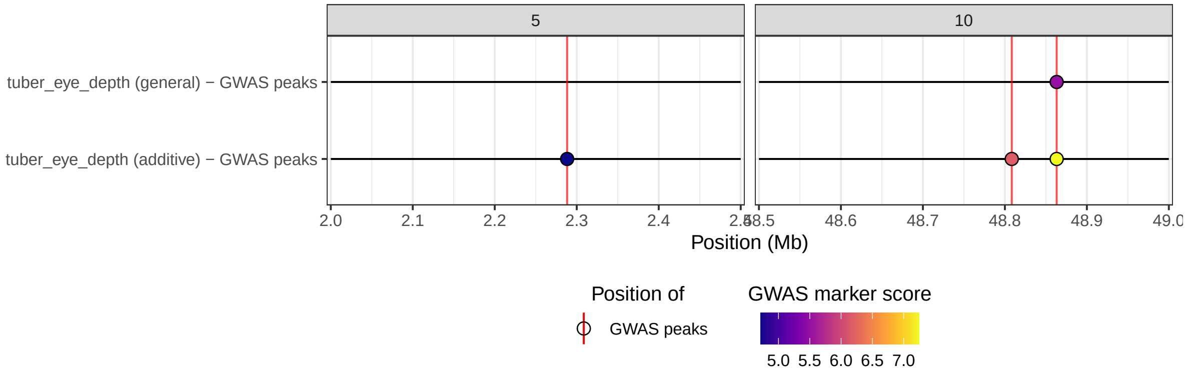

This example is also available online at https://plantandfoodresearch.github.io/hidecan/articles/web_only/gwaspoly_output.html.

Example 3 – adding custom data tracks

The data

In this third example, we show how we can add other data types than GWAS or DE results to a HIDECAN plot. For that, we will simulate some data that include GWAS results, significant regions from QTL mapping, and results from a differential methylation analysis.

We will start by simulating GWAS results for 500 markers spread across two chromosomes:

```
library(tibble)

sim_data <- list()
chroms <- c("Chromosome 1", "Chromosome 2")

set.seed(216)
n <- 500
sim_data$GWAS <- tibble(
  chromosome = sample(chroms, n, replace = TRUE),
```

```
  position = runif(n, 0, 60e6),
  score = -log10(runif(n, 0, 1))
)
```

We then create two QTL regions of interest, one on each chromosome. We need to give these regions a score, and we will give them a name as well to show on the plot:

```
sim_data$QTL <- tibble(
  chromosome = chroms,
  position = c(25e6, 37e6),
  score = c(3, 6),
  name = c("QTL 1", "QTL 2")
)
```

Finally, we will generate differential methylation for 500 genomic regions:

```
sim_data$DMR <- tibble(
  chromosome = sample(chroms, n, replace = TRUE),
  position = runif(n, 0, 60e6),
  score = -log10(runif(n, 0, 1))
)
```

Constructing the HIDECAN plot

In order to add these two new data types to the HIDECAN plot, we need to specify the aesthetics of these new tracks. In particular, we need to set:

- the label of each data type on the x-axis (e.g. 'GWAS peaks' for GWAS results),
- the colour of the vertical lines used to showcase the position of the genomic features,
- the shape of the points representing the genomic features,
- whether the name of the genomic features are displayed,
- the colour palette used for the genomic features' score.

We specify these by creating a named list that holds values for these parameters for each data type. For the QTL regions, we want to use a golden line, show the regions' name but not their score (hence the `fill_scale` element is `NULL` for `QTL_data`). For the differentially methylated regions, we use the 'rocket' palette from the `viridis` package to display their score.

```r
library(ggplot2)

# Setting up a colour palette for differential methylation score
dmr_palette <- scale_fill_viridis_c(
  option = "rocket",
  name = "DM score",
  guide = guide_colourbar(
    title.position = "top",
    title.hjust = 0.5
  )
)

# Aesthetics for the custom data types
plot_aes <- list(
  QTL_data = list(
    y_label = "QTL regions",
    line_colour = "darkgoldenrod2",
    point_shape = 18,
    show_name = TRUE,
    fill_scale = NULL
  ),

  DMR_data = list(
    y_label = "Diff. methylated regions",
    line_colour = "orchid",
    point_shape = 24,
    show_name = FALSE,
    fill_scale = dmr_palette
  )
)
```

Note that the names given to this list that indicate each data type (i.e. `QTL_data` and `DMR_data`) are completely arbitrary. What is important is that they need to match with the next part of the code.

Next, we will add an attribute to each input data-frame which specifies the aesthetics to be used in the plot; this is the part where we need to use the same labels as in our list of aesthetics:

```r
# Specify the data type of each table
attr(sim_data[["QTL"]], "aes_type") <- "QTL_data"
attr(sim_data[["DMR"]], "aes_type") <- "DMR_data"
```

Finally, we can generate the HIDECAN plot. The custom datasets are passed to `hidecan_plot()` via the `custom_list` argument. We can filter these datasets by score through the `score_thr_custom` argument; but note that the same threshold will be used to filter all datasets, so it might be preferable to filter the significant features beforehand and then set this threshold to 0. The list of aesthetics to use for each data type is passed to the function via the `custom_aes` argument:

```
hidecan_plot(
  gwas_list = sim_data[["GWAS"]],
  score_thr_gwas = 2,
  custom_list = list(sim_data[["QTL"]], sim_data[["DMR"]]),
  score_thr_custom = 2,
  custom_aes = plot_aes
)
```

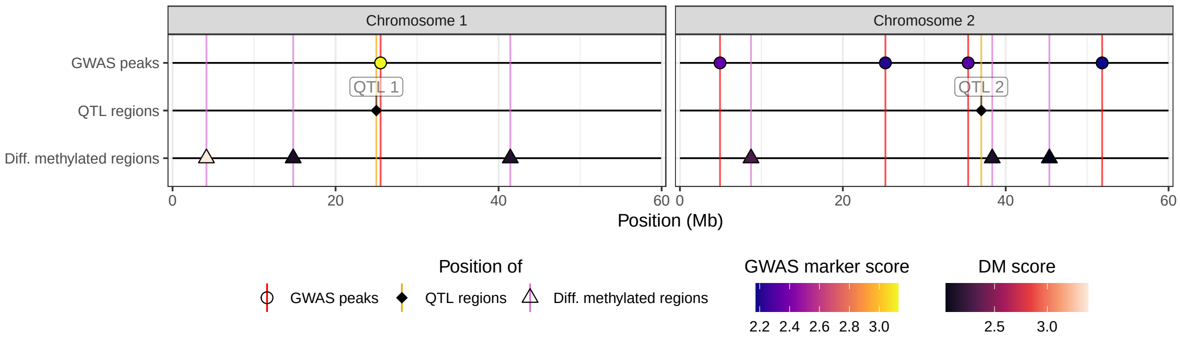

More information about adding custom data tracks to a HIDECAN plot is also available online at https://plantandfoodresearch.github.io/hidecan/articles/web_only/custom_tracks.html.

References

Angelin-Bonnet, Olivia, Susan Thomson, Matthieu Vignes, Patrick J. Biggs, Katrina Monaghan, Rebecca Bloomer, Kathryn Wright, and Samantha Baldwin. 2023. "Investigating the genetic components of tuber bruising in a breeding population of tetraploid potatoes." *BMC Plant Biol* 23 (1): 238. https://doi.org/10.1186/s12870-023-04255-2.

Rosyara, Umesh R., Walter S. De Jong, David S. Douches, and Jeffrey B. Endelman. 2016. "Software for genome-Wide association studies in autopolyploids and its application to potato." *Plant Genome* 9 (2). https://doi.org/10.3835/plantgenome2015.08.0073.
